# Origin of proton affinity to membrane/water interfaces

**DOI:** 10.1038/s41598-017-04675-9

**Published:** 2017-07-03

**Authors:** Ewald Weichselbaum, Maria Österbauer, Denis G. Knyazev, Oleg V. Batishchev, Sergey A. Akimov, Trung Hai Nguyen, Chao Zhang, Günther Knör, Noam Agmon, Paolo Carloni, Peter Pohl

**Affiliations:** 1 Institute of Biophysics, Johannes Kepler University Linz, 4040 Linz, Austria; 2 Institute of Inorganic Chemistry, Johannes Kepler University Linz, 4040 Linz, Austria; 3 A.N. Frumkin Institute of Physical Chemistry and Electrochemistry, Russian Academy of Sciences, Leninskiy pr. 31/4, Moscow, 119071 Russian Federation; 4 Moscow Institute of Physics and Technology, Institutsky lane, 9, 141700 Dolgoprudniy, Russian Federation; 5 National University of Science and Technology “MISiS”, Leninskiy pr. 4, Moscow, 119991 Russian Federation; 6 Computational Biomedicine (IAS-5 / INM-9) Forschungszentrum Jülich, 52425 Jülich, Germany, RWTH Aachen University, 52056 Aachen, Germany; 7 Institute of Chemistry, The Hebrew University of Jerusalem, Jerusalem, 9190401 Israel; 8 Department of Chemistry, University of Cambridge, Lensfield Rd, Cambridge, CB2 1EW United Kingdom

## Abstract

Proton diffusion along biological membranes is vitally important for cellular energetics. Here we extended previous time-resolved fluorescence measurements to study the time *and* temperature dependence of surface proton transport. We determined the Gibbs activation energy barrier Δ*G* ^‡^_r_ that opposes proton surface-to-bulk release from Arrhenius plots of (i) protons’ surface diffusion constant and (ii) the rate coefficient for proton surface-to-bulk release. The large size of Δ*G* ^‡^_r_ disproves that quasi-equilibrium exists in our experiments between protons in the near-membrane layers and in the aqueous bulk. Instead, non-equilibrium kinetics describes the proton travel between the site of its photo-release and its arrival at a distant membrane patch at different temperatures. Δ*G* ^‡^_r_ contains only a minor enthalpic contribution that roughly corresponds to the breakage of a single hydrogen bond. Thus, our experiments reveal an entropic trap that ensures channeling of highly mobile protons along the membrane interface in the absence of potent acceptors.

## Introduction

Proton production and consumption processes play a pivotal role for bioenergetics across all organisms^1^. Most of these processes involve proton diffusion at the cellular membrane/water interface^2, 3^. For instance, the synthesis of adenosine triphosphate (ATP), the free energy carrier in living systems, relies on two types of membrane-bound enzymes: proton pumps creating the transmembrane proton gradient and ATP synthases consuming this transmembrane potential to drive ATP synthesis^4, 5^. Strikingly, protons move extremely fast along lipid membranes^6, 7^: their lateral proton diffusivity is almost as large as in bulk water^7, 8^. This fast proton migration establishes an efficient link between these proton release and consumption sites^3, 9, 10^.

The long interfacial travel distance observed for protons implies that a substantial free energy barrier, Δ*G* ^‡^_r_, for proton release prevents the surface proton from readily equilibrating with its bulk counterparts^7, 11^. It allows placing regulatory proteins (uncoupling protein 4) at some distance from both ATP synthases and proton pumps on the inner mitochondrial membrane^12^. Due to the spatial separation the uncoupling protein cannot uncouple phosphorylation from proton pumping. However, the large Δ*G* ^‡^_r_ routes excessive protons along the membrane surface to the distant proton leak. In turn, the production of reactive oxygen species is decreased.

**Figure 1:**
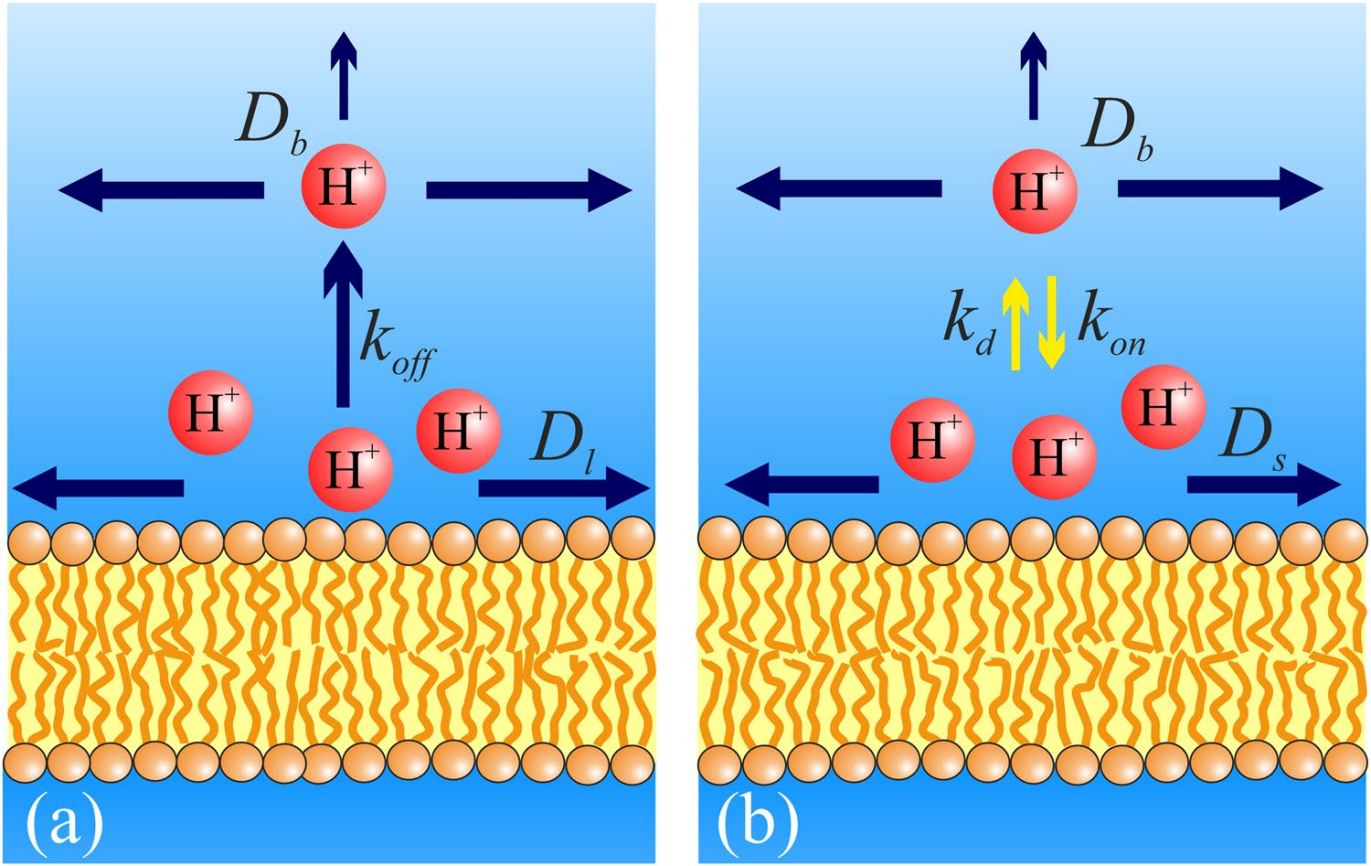
Schematic of the two different models for proton migration along the membrane surface. The non-equilibrium model (**a**) envisions proton diffusion within the confinement of the membrane hydration layers without the involvement of titratable residues on the surface. Proton surface-to-bulk release is thought to be irreversible (equation (1)). The quasi-equilibrium model (**b**) treats proton surface diffusion as a succession of jumps between titratable residues. The proton uptake and release reactions are in equilibrium. In the absence of real proton acceptors and donors, fictional moieties are assumed to take their place. Accordingly, their p*K*_a_ value is obtained as a fitting parameter of the model (equations (3–6)).

Δ*G* ^‡^_r_ values can be estimated from experiments in which protons are photo-released on a membrane area at distance *x* (tens of micrometers) from the observation patch^6, 7^. To explain the results, two different models have been proposed (Fig. 1): The first model assumes that proton uptake by the interface is not in equilibrium with proton surface-to-bulk release^13^, whereas the second assumes quasi-equilibrium between interfacial and bulk protons^14^.

The non-equilibrium model describes the proton concentration, *σ*, in the water layers adjacent to the membrane at time *t* as a function of both the interfacial (lateral, two dimensional) proton diffusion constant, *D*_l_, and the release rate coefficient, *k*_off_, from the membrane surface^13^:

1 $$\sigma (x,t)={\sigma }_{0}+\frac{{A}_{neq}}{4\pi {D}_{{\rm{l}}}t}\exp (-\frac{{x}^{2}}{4{D}_{{\rm{l}}}t})\exp (-t\,{k}_{{\rm{off}}})\,$$

Here *A*_neq_ is a measure of proton concentration increase (of the non-equilibrium model) and *σ*_0_ denotes the pre-existing proton concentration adjacent to the surface. Previously measured *k*_off_ values^13^ of about 0.5 s^−1^ can be used to estimate Δ*G* ^‡^_r_ ≈ 30 *k*_B_ *T*:

2 $${k}_{off}={\nu }_{0}\,\exp (-{\rm{\Delta }}{{\rm{G}}}_{{\rm{r}}}^{\ddagger}/{k}_{B}T),$$

where *T*, *k*_B_, and *ν*_0_ ≈ 10^13^ s^−1^ are the absolute temperature, the Boltzmann constant, and the universal transition state theory attempt frequency for rate processes at surfaces^15^.

The second, quasi-equilibrium model assumes that Δ*G* ^‡^_r_ can be computed from the bulk proton concentration, [H^+^]_bulk_, and σ as^16^: Δ*G* ^‡^_r_ = *k*_B_ *T* ln(σ/[H^+^]_bulk_). Surface and bulk protons are thought to be coupled over distance *L*_0_ (Fig. 1):

3 $$\sigma (x,t)={\sigma }_{0}+\frac{{A}_{eq}}{4\pi {D}_{{\rm{s}}}t}\exp (-\frac{{x}^{2}}{4{D}_{{\rm{s}}}t}){(1+{(\frac{\sqrt{\pi {D}_{{\rm{s}}}t}}{{L}_{0}})}^{\alpha })}^{-1}.$$

where *A*_eq_ is a measure of proton concentration increase (of the quasi-equilibrium model)^14^. Equation (3) is a much simplified version of the original model^17^. It has been obtained by assuming that the proton surface diffusion coefficient, *D*_s_, and the proton bulk diffusion coefficient, *D*_b_, are equal to each other. The dimensionality α = 1 for transversal proton motion holds for an ideal infinite plane, i.e. when the interfacial water layer width, *d* ~ 1 nm, for surface proton diffusion is much smaller than *L*_0_:

4 $${L}_{0}=d\,\exp ({{\rm{\Delta }}G}_{{\rm{r}}}^{\ddagger}/{k}_{B}T).$$

Assuming *L*_0_ = 170 µm^18^ yields Δ*G* ^‡^_r_ ≈ 12 *k*_B_ *T*.

The quasi-equilibrium model^17^ takes into account all titratable groups at the membrane surface. Each of them is thought to occupy surface area *A*_tg_ and to be characterized by proton binding and dissociation coefficients *k*_on_ and *k*_d_, respectively^18^:

5 $${L}_{0}=\frac{{k}_{{\rm{on}}}}{{k}_{{\rm{d}}}{N}_{{\rm{A}}}{A}_{tg}},$$

where *N*_A_ is Avogadro’s number. Setting the dwell time *t*_0_ of the proton within the interfacial water layer equal to be *d* ^2^/*D*_s_ times a Boltzmann factor depicting the delay in proton surface-to-bulk release, allows transforming equation (4)^14^:

6 $${t}_{0}=\frac{{d}^{2}\,}{{D}_{s}}\exp (\frac{{\rm{\Delta }}{G}_{r}^{\ddagger}}{{k}_{{\rm{B}}}T})=\frac{{L}_{0}d}{{D}_{{\rm{s}}}}$$

Thus with *k*_d_ = 1/*t*_0_, equations (5) and (6) imply that ${k}_{on}={N}_{{\rm{A}}}{A}_{tg}{D}_{s}/d$, namely a diffusion controlled association (*k*_on_ proportional to *D*_s_). For phosphatidylethanolamine bilayers equations (5) and (6) yield *t*_0_ ≈ 1 s and *L*_0_ ≈ 13 m, respectively, since *k*_on_/*k*_d_ and A_tg_ are equal to 10^9.6^ M^−1^ and 51 Å^2^, respectively^19, 20^. Phosphatidylcholine bilayers provide another example: *L*_0_ must be in the order of one micrometer and *t*_*0*_ in the order of 100 ns since *k*_d_ is roughly 7 orders of magnitude larger. However, neither one of the extremes has been observed experimentally: dwell time and diffusion span were in the order of one second and 100 micrometers, respectively, for both bilayers^7^. In consequence, the quasi-equilibrium model now rests on fictitious moieties (equation (5))^14^. Thus, *L*_0_, *D*_s_, and α are fitting parameters in equation (3).

One goal of the present work is to differentiate between the two theoretical models (equation (1) versus (3–6)) by means of measuring Δ*G* ^‡^_r_. The second goal is to establish whether this quantity contains a large entropic contribution, which could explain the affinity of the proton to the hydration water in the absence of a potent proton acceptor. In order to address these issues, we monitored the temperature dependence of proton diffusion kinetics along the surface of lipid bilayers.

## Results

**Figure 2:**
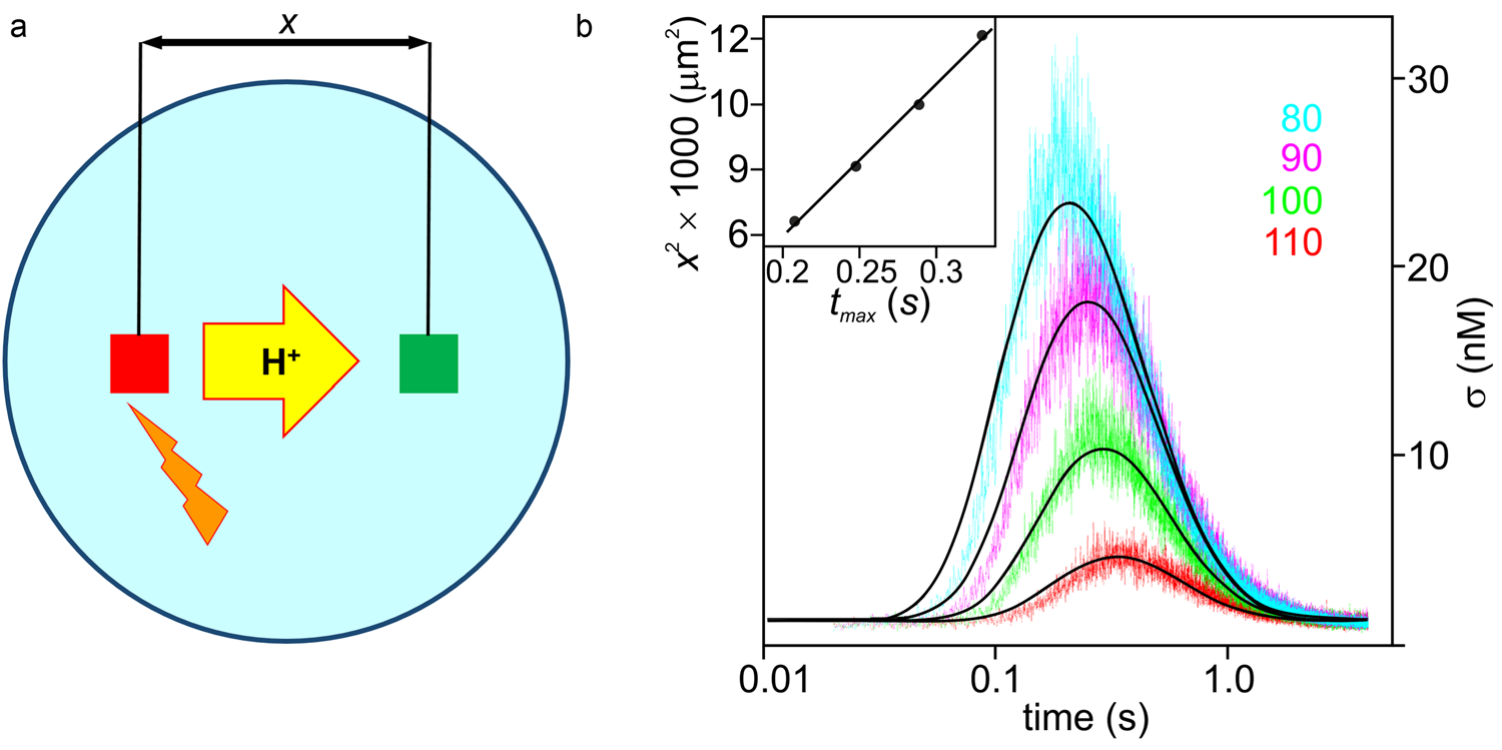
Monitoring proton surface diffusion. (**a**) The membrane bound caged protons were released by a UV flash from the area in the red square (10 × 10 µm^2^) and their arrival was observed as a change in fluorescence intensity in the green square (10 × 10 µm^2^). The light emitted by the lipid-anchored pH sensor fluorescein was collected using a 40x water immersion objective and a 515 nm high-pass filter. (**b**) The proton concentration σ adjacent to the membrane is monitored as a function of the time that elapsed after the flash at the indicated distances *x* (in µm) from the observation site (19 °C). σ has been calculated from the fluorescence intensity of membrane anchored fluorescein according to a calibration curve (Supplementary Fig. S1). It reaches it maximum at time *t*_max_, which according to equation (1) obeys: *t*_max _ = *x* ^2^/4*D* − *k*_off_ *t*_max_^2^. When the last term is small, *t*_max_ depends linearly on *x* ^2^, as shown in the inset. The colored traces are averaged data from at least 10 individual uncaging reactions each. The black lines represent a global fit to average traces at four distances of the non-equilibrium model. Therefore equation (1) was modified to take into account the finite sizes of release and detection zones (equation (S3), Supplementary Fig. S2). The global fit parameters, *D*_l_ = 5.1 × 10^−5^ cm^2^ s^−1^ and *k*_off_ = 2.3 s^−1^, are common to all curves, whereas the amplitude *A*_neq_ was allowed to vary (±15%).

We released the protons from a membrane bound caged compound^6^, by exposing a 10 × 10 µm^2^ membrane area to a UV flash (wavelength < 400 nm) (red square in Fig. 2a). We then recorded the time-dependent intensity changes in fluorescence of a lipid-anchored pH-sensitive dye (N-(fluorescein-5-thiocarbamoyl)-1,2-dihexadecanoyl-sn-glycero-3-phosphoethanolamine) at distance *x* from the release area that was excited by illumination at 488 nm (green square in Fig. 2a)^6, 7^. The resulting fluorescence decreases as protons reach the observation spot and then increases as they diffuse further away. The time between proton release and arrival increases with increasing *x* while the number of protons reaching the destination decreases (Fig. 2b). See Materials and Methods for additional detail.

**Figure 3:**
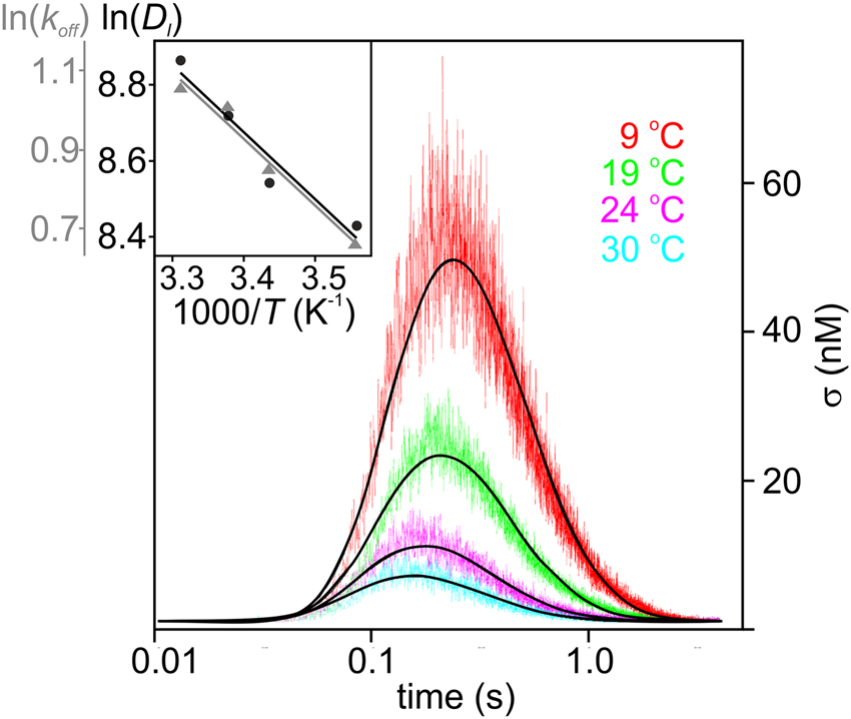
Kinetics of the proton concentration adjacent to the membrane surface at 80 µm from the release spot for different temperatures. The colored traces are averaged data from at least 10 individual release events each. At least three such averaged traces had been obtained at every temperature for four distances: 80, 90, 100 and 110 µm. The global fits of the non-equilibrium model (equation (S3)) to all traces at a given temperature is depicted as solid black lines. Inset: Temperature dependencies of the rate coefficient for proton surface-to-bulk release (*k*_off_, in units of s^−1^) and for the lateral diffusion constant (*D*_l_, in units µm^2^ s^−1^). The slopes correspond to Δ*H* ^‡^_l_ ≈ 5.9 ± 1.1 *k*_*B*_ *T* and Δ*H* ^‡^_r_ ≈ 5.7 ± 0.7 *k*_B_ *T*, whereas the intercepts with the y-axis are *A*_l_ = (3.3 ± 0.5) × 10^6^ µm^2^ s^−1^ and *A*_r_ = (8.1 ± 0.9) × 10^2^ s^−1^, respectively (compare equations (7) and (9)).

An increase in temperature enhances the probability of proton surface-to-bulk release, so that fewer protons arrive at the observation spot. At the same time, proton mobility increases, so that σ reaches its maximum earlier (Fig. 3). We obtain *D*_l_ and *k*_off_ from global fits to equation (S3) (black lines). Equation (S3) differs from equation (1) by taking into account the exact sizes of the proton release and the observation areas (see Supplement). An Arrhenius plot (inset, Fig. 3)

7 $${D}_{{\rm{l}}}={A}_{{\rm{l}}}\exp (\frac{-{\rm{\Delta }}{H}_{{\rm{l}}}^{\ddagger}}{{k}_{B}T}),$$

permits calculation of the activation enthalpy,$\,{\rm{\Delta }}{H}_{{\rm{l}}}^{\ddagger}$ = 5.9 ± 1.1 *k*_B_ *T*. It is roughly 20% larger than the experimental activation enthalpy of 4.3 *k*_B_ *T* for bulk proton mobility^21^. The pre-exponential factor *A*_l_ allows assessment of *ν*_0_ = 17 × 10^13^ s^−1^ using the Einstein relation (in two dimensions):

8 $${v}_{0}={A}_{{\rm{l}}}\frac{4}{{l}^{2}}\,,$$

where *l* = 2.8 Å is the O-O distance in liquid water across which the proton hops. *D*_l_ indicates that interfacial proton mobility is very close to its value in unbuffered bulk water^22^. *ν*_0_ is about 10 times larger than its commonly accepted value. Such an increment is sometimes taken as indicative for proton tunneling^23^. Indeed, the previously observed isotope effect for proton surface diffusion (about 8)^7^ is larger than for proton mobility in bulk water (ca. 1.5).

With this encouraging result, we proceed to analyze the proton release reaction:

9 $${k}_{off}={A}_{r}\,\exp (-\frac{{\rm{\Delta }}{H}_{{\rm{r}}}^{\ddagger}}{{k}_{B}T})$$

The Arrhenius plot (inset Fig. 3) gives Δ*H* ^‡^_r_ = 5.7 ± 0.7 *k*_B_ *T*. Interestingly, the activation enthalpies for proton motion perpendicular and parallel to the membrane are nearly identical. Thus, the small preexponential, *A*_r_, is responsible for the long retention time of protons on the membrane. From transition state theory we anticipate that *A*_r_ = *ν*_0_ exp(Δ*S* ^‡^_r_/*k*_B_), where Δ*S* ^‡^_r_ is the entropy of activation for proton release:

10 $$T{\rm{\Delta }}{{\rm{S}}}_{{\rm{r}}}^{\ddagger}={k}_{B}T\,\mathrm{ln}({A}_{r}/{\nu }_{0})=-26.1{k}_{B}T$$

where we have used *ν*_0_ = 17 × 10^13^ s^−1^ (equation (8)). This implies Δ*G* ^‡^_r_ = Δ*H* ^‡^_r_ −*T*Δ*S* ^‡^_r_ = 31.8 *k*_B_ *T*. The equation Δ*G* ^‡^_r_ = −*k*_B_ *T* ln(*k*_off_/*ν*_0_) yields a similar value (for 24 °C) attesting to the consistency of this analysis. It is therefore predominantly an *entropy effect* which opposes proton release from surface-to-bulk.

**Figure 4:**
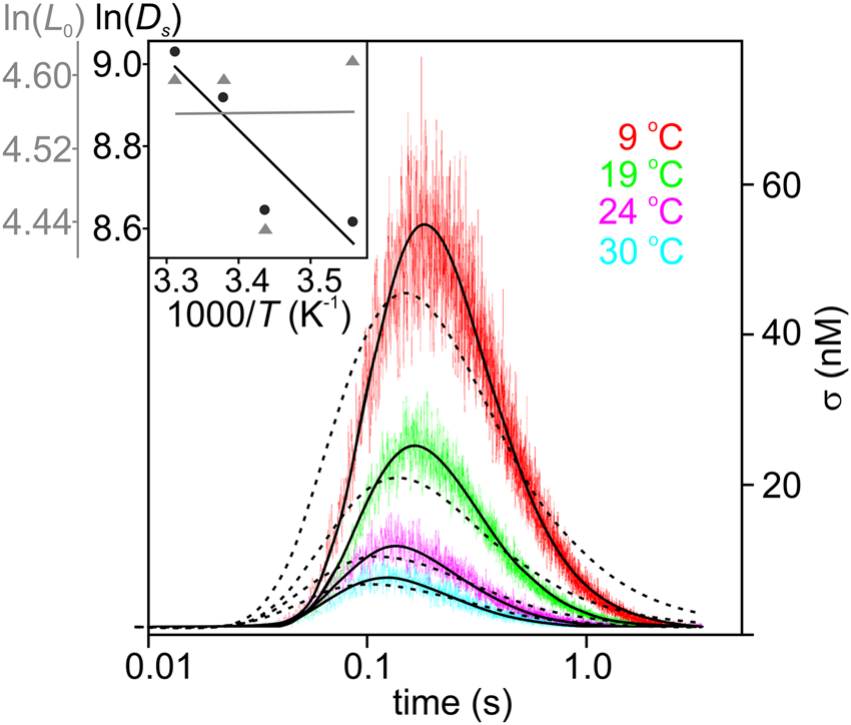
The quasi-equilibrium model (compare equation (3)**)** does not fit the data for *d* ≪ *L*_0_ (dashed line, for parameters see Supplementary Table S1) if the surface-to-bulk release reaction is assumed to be one dimensional (α = 1). As in the case of the non-equilibrium model we took the finite sizes of proton release and detection areas into account (equation (S4)). The experimental data (colored lines) were taken from Fig. 3. The assumption α = 3 (solid black lines) yielded a satisfactory fit. Inset: The global fit of the quasi-equilibrium model (α = 3) to the traces measured at 80, 90, 100, and 110 µm produced a temperature independent *L*_0_ of roughly 100 µm. The corresponding Arrhenius plot of *D*_s_ (in units of µm^2^ s^−1^) revealed Δ*H* ^‡^_l_ = 5.8 ± 2.0 *k*_*B*_ *T* and a pre-exponential factor *A*_*l*_ = (3.3 ± 0.5) × 10^6^ µm^2^ s^−1^. These values are essentially identical to those obtained from the non-equilibrium model in Fig. 3.

Next we tested whether the quasi-equilibrium model, equation (3), satisfactorily describes the data. α = 1 did not fit the data, although we accounted for the exact sizes of the proton release and measurement spots (equation (S4)). Augmenting α to 2 also resulted in an unsatisfactory fit. Solely upon setting α = 3 were we able to fit the quasi-equilibrium model to the data (Fig. 4).

That is, we globally fitted equation (S4) to several σ(*t*) profiles that were measured at distances *x* = 80, 90, 100, and 110 µm (Fig. 2). We repeated the procedure at four different temperatures and thereby extracted both *D*_s_ and *L*_0_ from the experiments (Supplementary Table S1). *L*_0_ showed little variation with temperature being always roughly equal to ~100 µm (Fig. 4, inset). We find Δ*G* ^‡^_r_ ≈ 0 and *d* = *L*_0_ ≈ 100 µm according to equation (4) (Fig. 4, inset). The temperature-independent *L*_0_ means that 1/*t*_0_ of equation (6) has the same temperature dependence (Supplementary Fig. S3) as *D*_s_.

The large *d* value is in stark contrast to the originally assumed value of only 1 nm, contrasting with the requirement that *d* ≪ *L*_0_. *d* ≈ *L*_0_ would be consistent with a three dimensional proton escape reaction (α = 3) (Fig. 4). However, augmenting α above 1 renders the mathematical description of the quasi-equilibrium model questionable. For α = 3, the dimensionality of the membrane degenerates to 0. As we show in our Supplement, for α = 2 equation (3) represents an approximation of equation (1), i.e. it no longer describes quasi-equilibrium (Supplementary Fig. S4). It is thus not surprising that the fitting parameters *D*_s_ and 1/*t*_0_ of the non-equilibrium model are close to those of the equilibrium model, *D*_l_ and *k*_off_, respectively (Supplementary Table S1). Accordingly, also the pre-exponents of the corresponding Arrhenius plots are close to each other (Figs 3, 4 and S3). We calculated them by substituting *D*_l_ for *D*_s_ in equation (7).

The fitting result Δ*G* ^‡^_r_ ≈ 0 (Fig. 4, inset) suggests the highly unrealistic scenario of a barrier-free proton escape. Proton hopping requires breaking of hydrogen bonds, so that Δ*H* ^‡^_r_ must be (i) at least as large as its counterpart in bulk water, i.e. ≈ 4.3 *k*_B_ *T* ^21^ or (ii) equal to the corresponding value for hopping along the membrane water interface, i.e. ≈ 5.8 *k*_B_ *T* (Fig. 4). If so, *T*Δ*S* ^‡^_r_ must adopt a similar value. A positive entropic energy term suggests that protons prefer the bulk solution over the more hydrophobic water/membrane interface. This is difficult to reconcile with the observation that an excess proton disturbs the tetrahedral bulk water structure and thus preferentially accommodates close to hydrophobic interfaces^24^.

Since the simplified version of the quasi-equilibrium model (equations (3–6)) did not describe the experiment, we abandoned the assumption of *D*_s_ and *D*_b_ equality. Instead, we repeated our analysis with the original model^17^. However, the corresponding analysis (equation (S5)) only revealed a satisfactory accuracy of the global fit (with three independent parameters: *L*_0_, *D*_s_, *D*_b_) to the experimental data for negligibly small *D*_s_ (i.e. *D*_s_ ≪ *D*_b_) (Supplementary Fig. S5). To compensate for the lack of proton surface diffusion, the model returned large *D*_b_ values (Supplementary Table S1). However, buffer molecules must have carried the majority of the bulk protons in our system, so that much smaller values were expected. This is evident from the fact that the buffer capacity (~50 µM) in our experiments exceeded the bulk proton concentration (1 nM) by more than four orders of magnitude. Thus *D*_b_ ~ 500 µm² s^−1^ was expected at room temperature instead of the calculated value of 12500 µm² s^−1^. Together with the temperature independent *L*_0_ (Supplementary Fig. S5), this observation strongly argues against the quasi-equilibrium model.

## Discussion

We observed proton migration along the membrane-water interface between two membrane patches. The rise and the decay of the fluorescence signal agree with a simple model, in which protons diffuse laterally on the two-dimensional surface of the membrane (diffusion coefficient *D*_l_), detaching from it slowly and irreversibly (rate coefficient *k*_off_) (equation (1)). *D*_l_ and *k*_off_ were in excellent agreement with previous studies where protons have similarly been released from a membrane adsorbed caged compound^6, 7^. The incompatibility of the large *D*_l_ value with proton jumps along titratable interfacial moieties^7^ raised the question concerning the origin of the high proton affinity to membranes. How are protons retained on the surface for so long (seconds) if not by attraction to titratable groups? The small value of *k*_off_ suggests a large free-energy barrier (Δ*G* ^‡^_r_ > 30 *k*_B_ *T*) for proton release, which is unphysically large if most of it is enthalpic. To help decipher this enigma, we have conducted a detailed temperature-dependent study of the membrane proton transport process.

The temperature dependence of both *D*_l_ and *k*_off_ (equation (1)) is a major result of our work that sheds light on the mechanism of proton conduction along membrane-water interfaces. Their Arrhenius plots reveal essentially identical enthalpies of activation for protons moving parallel and perpendicular to the membrane. Moreover, this enthalpy cost is not large – approximately the strength of a single water-water hydrogen bond, as in the Grotthuss mechanism for proton mobility^25^. Consequently, high energy binding forces are not involved in keeping the proton at the surface.

The intercepts of the Arrhenius plots show that the major contribution to Δ*G* ^‡^_r_ is entropic, and not enthalpic. The proton enjoys considerably larger entropy at the interface than in the bulk^24^. For example, there may be an enhanced probability for the H_5_O_2_^+^ cation at the interface, with the proton delocalizing between two water oxygens. Such a possibility is suggested e.g. from the infrared spectrum of H_5_O_2_^+^ attached to a benzene molecule^26^ or the distribution of heteropolyanions at the air-water interface^27^. In contrast, the dominant species in the bulk is the hydronium ion, H_3_O^+^, which forms exceedingly strong hydrogen-bonds in its first solvation shell^28^, restructuring the water network around it. Thus, the entropy of the water solvent will reduce once a proton moves from the interface to the bulk.

Proton binding to aqueous buffer molecules should also be considered^7^. However, it is important to note that buffer molecules do not contribute to *D*_l_ but only to *D*_b_^6^. This follows from the simple considerations that (i) proton release from buffer molecules is much slower than H_3_O^+^ dissociation due to the higher pK_a_ value of buffer molecules and (ii) the bulkier buffer molecules perform diffusion in all three dimensions. The hopping along hydrogen bonded surface water molecules also explains the observation that *D*_l_ is very close to the diffusion coefficient of protons in pure water.

Conceivably, water structuring at the membrane interface also contributes to the entropic nature of proton membrane affinity. Evidence for the non-random orientation of interfacial water molecules has been obtained by (a) phase-sensitive vibrational sum frequency generation spectroscopy^29^ and (b) measurements of membrane dipole potential^30–32^. Aligned water molecules are thought to contribute roughly 50% to the total value of membrane dipole potential of about 220–250 mV^30, 32^. Bilayers from (i) phospholipids with or without conventional headgroups (like ethanolamine, choline, or glycerol headgroups) or (ii) lipids that do not contain the anionic phosphate moiety, have similar dipole potentials^32^ suggesting that charged moieties are not required to orient water dipoles. This notion is supported by reports about a net orientation of water molecules at the interface to alkene^33^ or to other hydrophobic interfaces^34^. It is also in line with the finding that titratable residues are not required for interfacial proton migration^7^.

Mechanistic insight about how a preferential alignment of water dipoles normal to the membrane surface promotes proton diffusion parallel to the surface awaits discovery by molecular dynamics simulations. One possibility would be that water dipole orientation toward the interface^11, 35^ electrostatically favors proton movement in that direction while disfavoring surface-to-bulk release. Another explanation may be that hydrated excess protons create their own water wires parallel to the membrane boundary. Such an effect has been observed in silico for proton transport through a hydrophobic nanotube^36^. It could explain why Δ*G* ^‡^_r_ is so much larger than the previously calculated free energy difference Δ*G* for passing from close proximity of the phosphate moieties to the bulk. Multistate empirical valence bond (MS-EVB) calculations^37^ and classical molecular dynamic calculations using a HYDYN protocol^35^ resulted in Δ*G* ~ 8 *k*_B_ *T* and Δ*G* = 5 *k*_B_ *T*, respectively. They are in quantitative agreement with equilibrium experiments on DOPC^38^, which suggested a 100 fold increase of proton concentration at the surface, i.e Δ*G* ~ 4–5 *k*_B_ *T*. In contrast to Δ*G*, Δ*G* ^‡^_r_ does not allow conclusions about the difference between surface and bulk pH.

Unlike the non-equilibrium model, the quasi-equilibrium model does not properly describe the temperature dependence of the proton release reaction. Its simplified mathematical version^14^ returns an incredibly large thickness of the near-membrane water layers of ~100 µm. The mathematically more involved, original version^17^ nullifies *D*_s_ and renders *D*_b_ incredibly large (see Supplementary Table S1). Both versions nullify Δ*G* ^‡^_r_, while producing Δ*S* ^‡^_r_ with the wrong (positive) sign, which is quite implausible. The quasi-equilibrium fails because it is rooted on the assumption of an enthalpic attraction of the surface proton to the membrane surface. The disguise by cosmetic adaptations - as represented by abandoning the p*K*_a_ values of real titratable moieties and substituting them for p*K*_a_ values of fictitious moieties^14^ - cannot repair the principal misconception: the protons are not held at the membrane surface by covalent bonds, but they are captured by an entropic trap. The trap is provided by interfacial water, along which the proton migrates. This strips titratable moieties from the position to govern interfacial proton mobility by proton uptake or release reactions^7^. Occasionally a proton may be lost to these moieties or born by them, but since there are so many protons released at the surface in our experiments, the overall proton mobility is little affected by their presence. The conclusion holds for micrometer-sized objects, like the planar bilayer studied here, as well as for the much smaller lipid vesicles or nanodiscs. It is equally valid for the pump-probe approach used in the current study, as well for equilibrium experiments. In any case, the residence time of a proton that is covalently bound to a titratable moiety does not reflect the mobility of all the other interfacial protons and hence, it cannot be used to calculate *D*_s_. It is thus not surprising that attempts to calculate *D*_s_ from equilibrium protonation kinetics of fluorescent surface dyes^38, 39^ severely underestimated the mobility of the surface proton^6, 7, 40^ (compare also Fig. 1).

We conclude that the low proton acceptability of water does not exclude interfacial water wires from acting as “proton railways”. This mechanism markedly differs from the concept of titratable lipid moieties acting as proton collecting antennae^18^. By dissecting Δ*G* ^‡^_r_ into entropic and enthalpic contributions, we were able to show that only a minor part of proton’s surface affinity is due to energetic attraction to the interface. Both proton movement along and perpendicular to the membrane requires breaking of hydrogen bonds. The energy associated with that process does not depend on the directionality of proton movement, while the entropy increases substantially as the proton moves from the interface to the bulk, and this now appears to be a key factor in membrane energetics.

## Materials and Methods

The *Experimental setup* has been described previously^6, 7^. In brief, horizontal planar lipid bilayers were formed from a solution of 20 mg 1,2-dioleoyl-sn-glycero-3-phosphocholine (DOPC, Avanti Polar Lipids, Alabama) in 1 ml *n*-decane (Sigma-Aldrich, Missouri) in a 200–300 µm wide aperture of a Teflon septum. The solution contained ~1 mol % of the pH-sensor fluorescein that was covalently linked to N-(fluorescein-5-thiocarbamoyl)-1,2-dihexadecanoyl-sn-glycero-3-phosphoethanolamine (Fluorescein DHPE (FPE), ThermoFisher, Massachusetts), and a caged-proton compound, 6,7-dimethoxycoumarin-4-yl)methyl diethyl phosphate, synthesized as previously described^41^. Protons were released by UV light pulse (<400 nm) emitted by a xenon flash lamp that was focused onto a 10 × 10 µm^2^ membrane patch. The FPE fluorescence was excited by illuminating another 10 × 10 µm^2^ large membrane area using 488 nm radiation from a second xenon lamp (150 W). The emitted light passed a 515 nm high-pass filter and was measured by a photomultiplier. Proton surface concentrations were calculated from the fluorescence intensities with the help of a calibration curve that depicted the fluorescence intensity (normalized to peak fluorescence intensity) as a function of bulk pH. We measured that curve in equilibrium. The procedure ignores any pH difference that may have existed between bulk and the bilayer surface. The buffer consisted of 10 mM KCl and 0.1 mM Capso. pH was adjusted to 9.0.

## Electronic supplementary material

Supporting Information

## References

[1] Nicholls, D. G. & Ferguson, S. *Bioenergetics*. 4'th edn. (Academic Press, 2013).

[2] Williams RJP (1988). Proton circuits in biological energy interconversions. Annu. Rev. Biophys. Biophys. Chem..

[3] Heberle J, Riesle J, Thiedemann G, Oesterhelt D, Dencher NA (1994). Proton migration along the membrane surface and retarded surface to bulk transfer. Nature.

[4] Wraight CA (2006). Chance and design - Proton transfer in water, channels and bioenergetic proteins. Biochim. Biophys. Acta.

[5] Okazaki K, Hummer G (2013). Phosphate release coupled to rotary motion of F_1_-ATPase. Proc. Natl. Acad. Sci. USA.

[6] Serowy S (2003). Structural proton diffusion along lipid bilayers. Biophys. J..

[7] Springer A, Hagen V, Cherepanov DA, Antonenko YN, Pohl P (2011). Protons migrate along interfacial water without significant contributions from jumps between ionizable groups on the membrane surface. Proc. Natl. Acad. Sci. USA.

[8] Alexiev U, Mollaaghababa R, Scherrer P, Khorana HG, Heyn MP (1995). Rapid long-range proton diffusion along the surface of the purple membrane and delayed proton transfer into the bulk. Proc. Natl. Acad. Sci. USA.

[9] Öjemyr LN, Lee HJ, Gennis RB, Brzezinski P (2010). Functional interactions between membrane-bound transporters and membranes. Proc. Natl. Acad. Sci. USA.

[10] Sandén T, Salomonsson L, Brzezinski P, Widengren J (2010). Surface-coupled proton exchange of a membrane-bound proton acceptor. Proc. Natl. Acad. Sci. USA.

[11] Zhang C (2012). Water at hydrophobic interfaces delays proton surface-to-bulk transfer and provides a pathway for lateral proton diffusion. Proc. Natl. Acad. Sci. USA.

[12] Klotzsch E (2015). Superresolution microscopy reveals spatial separation of UCP4 and F_0_F_1_-ATP synthase in neuronal mitochondria. Proc. Natl. Acad. Sci. USA.

[13] Agmon N, Gutman M (2011). Bioenergetics: Proton fronts on membranes. Nat. Chem..

[14] Medvedev ES, Stuchebrukhov AA (2013). Mechanism of long-range proton translocation along biological membranes. FEBS Lett..

[15] Barth JV, Brune H, Fischer B, Weckesser J, Kern K (2000). Dynamics of surface migration in the weak corrugation regime. Phys. Rev. Lett..

[16] Medvedev ES, Stuchebrukhov AA (2014). Mechanisms of generation of local ΔpH in mitochondria and bacteria. Biochemistry (Moscow).

[17] Medvedev ES, Stuchebrukhov AA (2006). Kinetics of proton diffusion in the regimes of fast and slow exchange between the membrane surface and the bulk solution. J. Math. Biol..

[18] Georgievskii Y, Medvedev ES, Stuchebrukhov AA (2002). Proton transport via the membrane surface. Biophys. J..

[19] Gutman M, Nachliel E (1990). The dynamic aspects of proton transfer processes. Biochim. Biophys. Acta.

[20] McIntosh TJ, Simon SA (1986). Area per molecule and distribution of water in fully hydrated dilauroylphosphatidylethanolamine bilayers. Biochemistry.

[21] Agmon N (1996). Hydrogen bonds, water rotation and proton mobility. J. Chim. Phys. Phys. Chim. Biol..

[22] Glietenberg D, Kutschker A, Vonstack. M (1968). Diffusion Coefficient of Protons in Aqueous Solutions of Some Salts. Ber. Bunsenges. Phys. Chem..

[23] Garcia-Viloca M, Gao J, Karplus M, Truhlar DG (2004). How enzymes work: analysis by modern rate theory and computer simulations. Science.

[24] Agmon N (2016). Protons and hydroxide ions in aqueous systems. Chem. Rev..

[25] Agmon N (1995). The Grotthuss Mechanism. Chem. Phys. Lett..

[26] Wang H, Agmon N (2015). Protonated water dimer on benzene: standing Eigen or crouching Zundel?. J. Phys. Chem. B.

[27] Bera MK, Antonio MR (2016). Aggregation of Heteropolyanions Implicates the Presence of Zundel Ions Near Air-Water Interfaces. ChemistrySelect.

[28] Markovitch O, Agmon N (2007). Structure and energetics of the hydronium hydration shells. J. Phys. Chem. A.

[29] Chen XK, Hua W, Huang ZS, Allen HC (2010). Interfacial Water Structure Associated with Phospholipid Membranes Studied by Phase-Sensitive Vibrational Sum Frequency Generation Spectroscopy. J. Am. Chem. Soc..

[30] Gawrisch K (1992). Membrane dipole potentials, hydration forces, and the ordering of water at membrane surfaces. Biophys. J..

[31] Pohl P, Rokitskaya TI, Pohl EE, Saparov SM (1997). Permeation of phloretin across bilayer lipid membranes monitored by dipole potential and microelectrode measurements. Biochim. Biophys. Acta.

[32] Peterson U (2002). Origin of membrane dipole potential: contribution of the phospholipid fatty acid chains. Chem. Phys. Lipids.

[33] Strazdaite S, Versluis J, Bakker HJ (2015). Water orientation at hydrophobic interfaces. J. Chem. Phys..

[34] Iuchi S, Chen H, Paesani F, Voth GA (2009). Hydrated Excess Proton at Water−Hydrophobic Interfaces. J. Phys. Chem. B.

[35] Wolf MG, Grubmüller H, Groenhof G (2014). Anomalous surface diffusion of protons on lipid membranes. Biophys. J..

[36] Peng Y, Swanson JM, Kang SG, Zhou R, Voth GA (2015). Hydrated Excess Protons Can Create Their Own Water Wires. J. Phys. Chem. B.

[37] Yamashita T, Voth GA (2010). Properties of hydrated excess protons near phospholipid bilayers. J. Phys. Chem. B.

[38] Brändén M, Sandén T, Brzezinski P, Widengren J (2006). Localized proton microcircuits at the biological membrane-water interface. Proc. Natl. Acad. Sci. USA.

[39] Xu L, Öjemyr LN, Bergstrand J, Brzezinski P, Widengren J (2016). Protonation dynamics on lipid nanodiscs: influence of the membrane surface area and external buffers. Biophys. J..

[40] Antonenko YN, Pohl P (2008). Microinjection in combination with microfluorimetry to study proton diffusion along phospholipid membranes. Eur. Biophys. J..

[41] Geißler D (2005). (Coumarin-4-yl)methyl esters as highly efficient, ultrafast phototriggers for protons and their application to acidifying membrane surfaces. Angew. Chem. Int. Ed..

