# Supplementary material for: Origin of proton affinity to membrane/water interfaces
Source: Sci Rep. 2017 Jul 3;7:4553. doi: 10.1038/s41598-017-04675-9 (PMC5495794; doi:10.1038/s41598-017-04675-9)
Supplement: Supplementary file 1 — Supporting Information [file 41598_2017_4675_MOESM1_ESM.pdf]

Supporting Information

Origin of proton affinity to membrane/water interfaces

Ewald Weichselbaum[1], Maria Österbauer[2], Denis G. Knyazev[1], Oleg V. Batishchev[3,4], Sergey A. Akimov[3,5], Trung Hai Nguyen[6], Chao Zhang[6,†], Günther Knör[2], Noam Agmon[7], Paolo Carloni[6], Peter Pohl[1,*]

[1]Institute of Biophysics, Johannes Kepler University Linz, 4040 Linz, Austria; [2]Institute of Inorganic Chemistry, Johannes Kepler University Linz, 4040 Linz, Austria; [3]A.N. Frumkin Institute of Physical Chemistry and Electrochemistry, Russian Academy of Sciences, Leninskiy pr. 31/4, Moscow, 119071, Russian Federation; [4]Moscow Institute of Physics and Technology, Institutsky lane, 9, 141700, Dolgoprudniy, Russian Federation; [5]National University of Science and Technology "MISiS", Leninskiy pr. 4, Moscow, 119991, Russian Federation; [6]Computational Biomedicine (IAS-5 / INM-9) Forschungszentrum Jülich, 52425 Jülich, Germany, RWTH Aachen University, 52056 Aachen, Germany; [7]Institute of Chemistry, The Hebrew University of Jerusalem, Jerusalem 91904, Israel

Proton release and detection occurs in an area of finite size P2

Proton diffusion constants and surface to bulk release constants P3

Calibration of pH measurements P4

Point diffusion model versus model with finite release and detection sites P5

Temperature dependence of proton dwell time (simplified quasi equilibrium model) P6

Approximating an exponential by a hyperbolic function P7

Testing the original (full) quasi equilibrium model P8

References P10

Proton release and detection occurs in an area of finite size

We modified diffusion equation (1) to account for the finite size of the proton release area:

$$\sigma_a(x',y',t)=\sigma_{a,0}+\frac{\sigma_{sq}}{4}\left(\operatorname{erf}\left(\frac{x'+h}{2\sqrt{Dt}}\right)-\operatorname{erf}\left(\frac{x'-h}{2\sqrt{Dt}}\right)\right)\left(\operatorname{erf}\left(\frac{y'+h}{2\sqrt{Dt}}\right)-\operatorname{erf}\left(\frac{y'-h}{2\sqrt{Dt}}\right)\right)\exp\left(-k_{off}t\right), \qquad \text{(S1)}$$

where σ_{sq} and $2h$ (= 10 µm in our setup) are the increment in proton concentration right after the excitation, and the side length of the excitation square, respectively. D is shorthand for D_l here. Erf(z) is the Gauss error function:

$$\operatorname{erf}(z)=\frac{2}{\sqrt{\pi}}\int_0^z e^{-\varsigma^2}d\varsigma$$

Since proton registration also occurs in an area of finite size, we integrate equation (S1) with the boundaries x-h and x+h and –h to h for the variables x' and y', respectively.

$$\sigma(x,t)=\frac{1}{4h^2}\int_{(x-h)}^{(x+h)}\int_{-h}^{h}\sigma_a(x',y',t)dx'dy' \qquad \text{(S2)}$$

Equation (S2) has the following analytical solution:

$$\begin{aligned}\sigma(x,t)=\sigma_0+A_n\exp\left(-k_{off}t\right)&\left[\operatorname{erf}\left(\frac{h}{\sqrt{Dt}}\right)h-\sqrt{\frac{Dt}{\pi}}\left(1-\exp\left(-\frac{h^2}{Dt}\right)\right)\right]\times\\ \times&\left[(x-2h)\operatorname{erf}\left(\frac{x-2h}{2\sqrt{Dt}}\right)+(x+2h)\operatorname{erf}\left(\frac{x+2h}{2\sqrt{Dt}}\right)-2x\operatorname{erf}\left(\frac{x}{2\sqrt{Dt}}\right)+\right.\\ &\left.+2\sqrt{\frac{Dt}{\pi}}\left(\exp\left(-\frac{(x+2h)^2}{4Dt}\right)+\exp\left(-\frac{(x-2h)^2}{4Dt}\right)-2\exp\left(-\frac{x^2}{4Dt}\right)\right)\right]\end{aligned}. \qquad \text{(S3)}$$

Similar considerations result in equation (S4) for the quasi equilibrium model (where D is shorthand for D_s here):

$$\begin{aligned}\sigma(x,t)=\sigma_0+A_e\left(1+\left(\sqrt{\frac{\pi Dt}{L_0}}\right)^{\alpha}\right)&\left[\operatorname{erf}\left(\frac{h}{\sqrt{Dt}}\right)h-\sqrt{\frac{Dt}{\pi}}\left(1-\exp\left(-\frac{h^2}{Dt}\right)\right)\right]\times\\ \times&\left[(x-2h)\operatorname{erf}\left(\frac{x-2h}{2\sqrt{Dt}}\right)+(x+2h)\operatorname{erf}\left(\frac{x+2h}{2\sqrt{Dt}}\right)-2x\operatorname{erf}\left(\frac{x}{2\sqrt{Dt}}\right)+\right.\\ &\left.+2\sqrt{\frac{Dt}{\pi}}\left(\exp\left(-\frac{(x+2h)^2}{4Dt}\right)+\exp\left(-\frac{(x-2h)^2}{4Dt}\right)-2\exp\left(-\frac{x^2}{4Dt}\right)\right)\right]\end{aligned} \qquad \text{(S4)}$$

Table S1 Proton diffusion constants and surface-to-bulk release constants - determined by fitting of equations. (S3 – S5) to the experimental data

Temperature	**9 °C**	**19 °C**	**24 °C**	**30 °C**
**Non-equilibrium model**				
D_l (μm^2 s^{-1})	4584	5126	6115	7065
k_{off} (s^{-1})	1.9	2.3	2.7	2.9
**Simplified quasi-equilibrium model (α = 1)**				
D_s (μm^2 s^{-1})	6449	6881	8188	8893
L_0 (μm)	18.0	14.7	14.1	12.2
**Simplified quasi-equilibrium model (α = 3)**				
D_s (μm^2 s^{-1})	5538	5690	7464	8330
L_0 (μm)	101	84	99	99
**Original (full) quasi-equilibrium model**				
D_s (μm^2 s^{-1})	0.001	0.001	0.001	0.001
D_b (μm^2 s^{-1})	9460	10124	12452	14038
L_0 (μm)	14.0	12.4	15.5	14.1

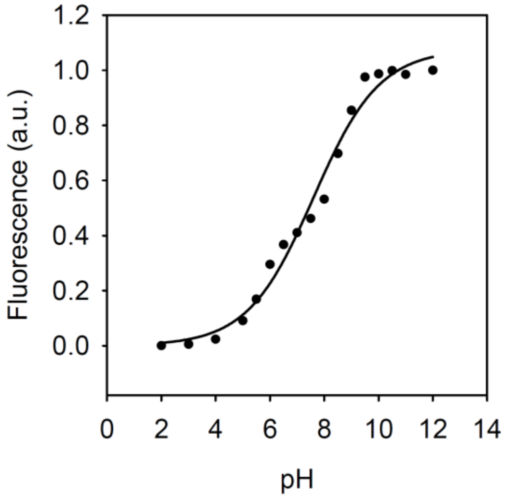

Fig. S1 Calibration curve. We measured the equilibrium fluorescence intensity of a lipid bilayer containing lipid-anchored fluorescein as a function of bulk pH. Here values normalized to peak fluorescence intensity are shown. The data are used to calculate proton concentration adjacent to the surface. The procedure ignores any pH difference that may have existed between bulk and the bilayer surface. The buffer consisted of 10 mM KCl and 0.1 mM Capso.

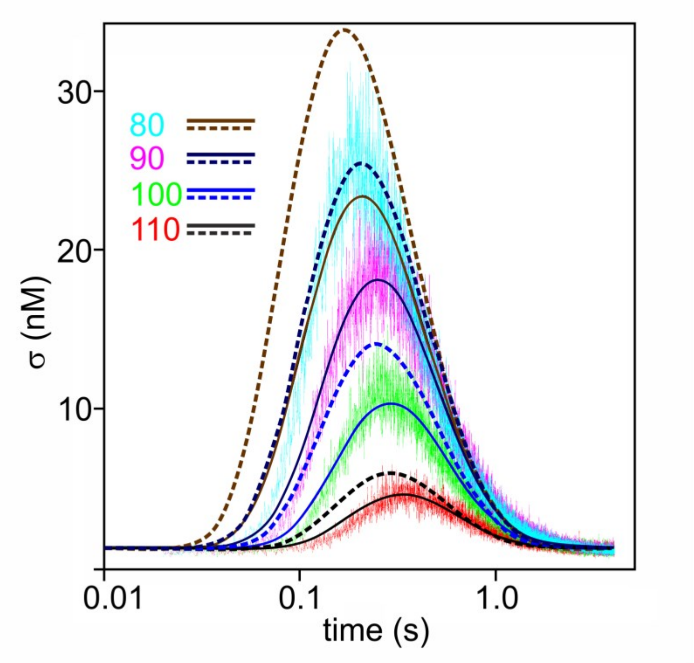

Fig. S2. Effect of the finite sizes of proton release and detection areas. The figure reproduces the data shown in Fig. 2b: Equation (S3) was globally fitted (solid lines) to measured proton concentrations that were detected in observation areas at 80 (cyan), 90 (magenta), 100 (green), and 110 (red) μm from the release area. Inserting the retrieved parameters D_l and k_{off} into equation (1) (models point-like proton source and point-like detection area) yielded the dashed lines.

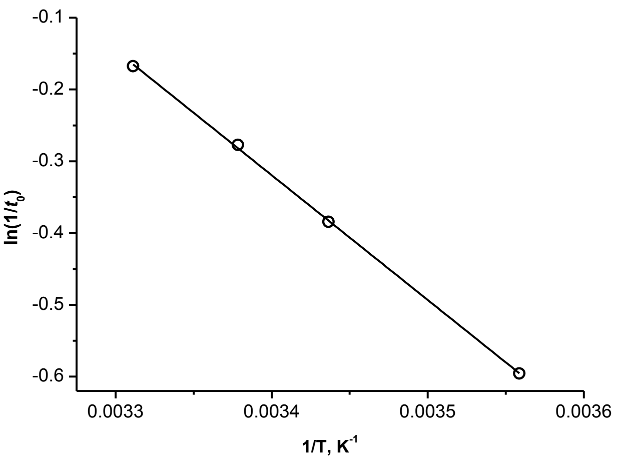

Fig. S3. Temperature dependence of $1/t_0$ (simplified quasi-equilibrium model). Assuming α = 3 and d = 100 μm allowed fitting of equation (S4) to the experimental data. From the linear fit of the Arrhenius plot $\ln(k_d) = (5.6 \pm 0.1) - (1739 \pm 21)\, 1/T$, we obtain $\Delta H_r^{\ddagger} = 5.9 \pm 0.1\ k_B T$ and $A_r = (2.7 \pm 0.1) \times 10^2\ s^{-1}$.

Approximating an exponential by a hyperbolic function

When $\alpha = 2$, equation (3) in the main text becomes an approximate form for equation (1), with the exp(-x) approximated as 1/(1+x). This simple result is derived as follows:

For small x range, an exponential may be approximated by a 1st order Taylor expansion, $\exp(-x) \approx 1-x$. This is good only near the origin (compare black & red lines in Fig. S4). Approximation of a hyperbolic function by a 1st order Taylor series gives: $1/(1+x) \approx 1-x$. Combining the two equations results in: $\exp(-x) \approx 1/(1+x)$, which is an improved approximation for the exponential function, see green line. Thus $\sigma(t,x)$ from equation (3) is an approximation to equation (1) with $k_{\mathrm{off}} = \pi D/L_0^2$. Compared with equation (6) in the main text we get $d \approx L_0$, as concluded in the text. Thus the quasi-equilibrium result (modified to have α=2) agrees with the data only with parameters that render it numerically similar to the irreversible model.

Parenthetically, we note that to get a more uniform convergence over an interval, one may introduce a small fudge-factor, e.g. $\exp(-x) \approx 1/(1+1.1x)$ (see blue line in Fig. S4). In comparison, the function $1/(1+x^{0.5})$ is a very poor approximation for an exponential, even after including some fudge factor, e.g. $1/(1+0.5x^{0.5})$ (see dashed magenta line).

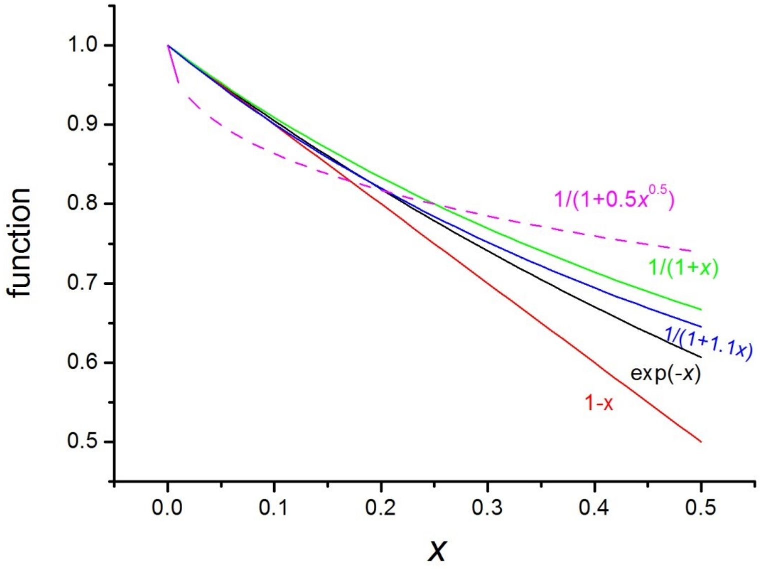

Fig. S4. Approximating an exponential by a hyperbolic function. For explanation see text.

Testing the original (full) quasi-equilibrium model.

We took equations 6, 20, 25, 33-37 of [1] to formulate equation (S5), which we then globally fitted to all experimental data at a given temperature. Equation (S5) considers 3 unknown parameters: D_s, D_b, L_0. It assumes a circular observation area with radius r_0 = 5 µm. At time t = 0 the excess proton is localized in a half-sphere, i.e. it partially adsorbs to the membrane (z = 0) and starts diffusing in the x, y, and z directions. The distance between release and observation areas is dubbed r_{obs}. For convenience the expressions $\beta = 1 - \left(D_s / D_b\right)$ and $t_0 = L_0^2 / D_b$ are used:

$$\sigma(r,t) = \int_0^\infty q dq J_0(qr)\left(G_{cut}(q,t) + G_{pole}(q,t)\right),$$

$$G_{cut}(q,t) = \frac{e^{\left(-\frac{1}{4}q^2 r_0^2\right)}}{2\pi L_0} e^{-D_b t q^2} \left(\frac{2}{\pi} \int_0^\infty \frac{e^{-\frac{t}{t_0}\xi^2} \xi^2 d\xi}{\xi^2 + \left(\xi^2 + \beta L_0^2 q^2\right)^2} \right), \tag{S5}$$

$$G_{pole}(q,t) = \frac{e^{\left(-\frac{1}{4}q^2 r_0^2\right)}}{2\pi L_0} \frac{\sqrt{1+4\beta L_0^2 q^2} - 1}{\sqrt{1+4\beta L_0^2 q^2}} \exp\left[-\frac{t}{2t_0}\left(\sqrt{1+4\beta L_0^2 q^2} - 1\right) - D_s t q^2 \right]$$

where J_0 is the Bessel function.

Equation (S5) was derived assuming a significant contribution of surface diffusion to proton flux [1]. This is in marked contrast to our fitting result that D_s approaches zero (Fig. S5). The quasi-equilibrium model foresees a constant exchange of bulk protons with surface protons during their travel parallel to the membrane ($L_0 \approx$ 14 µm). This explains why equation (S5) may fit the data – very much in contrast to the equation of true 3D bulk diffusion [2].

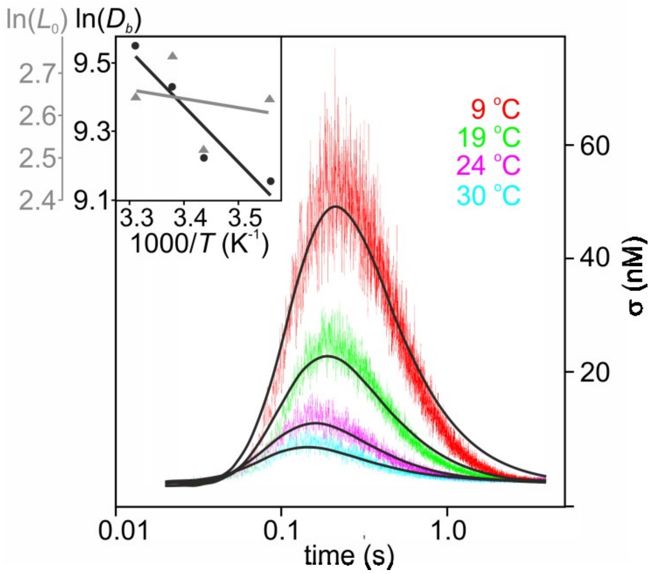

Fig. S5. Fit of the original (full) quasi equilibrium model to data (σ) measured at a distance of 80 μm from the release area at different temperatures (data were taken from Fig. 2). The best fit was obtained for $D_s \ll D_b$ (i.e. $D_s \approx 0$). Inset: Temperature dependence of D_b (in μm^2 s^{-1}) and L_0 (in μm). From the linear fit of the Arrhenius plot ln(D_b) = (15 ± 1) – (1637 ± 415) 1/T, we obtain $\Delta H_1^{\ddagger} = 6 \pm 1$ $k_B T$ and A_1= (3.1±0.2)×10^6 μm^2 s^{-1}. v_0 amounts to (1.6±0.1)×10^{14} s^{-1} according to equation (8). L_0 is independent on temperature. The pre-exponential factor d is equal to 20 ± 13 μm.

References

1 Medvedev, E. S. & Stuchebrukhov, A. A. Kinetics of proton diffusion in the regimes of fast and slow exchange between the membrane surface and the bulk solution. *J Math Biol* **52**, 209-234 (2006).

2 Serowy, S. *et al.* Structural proton diffusion along lipid bilayers. *Biophys. J* **84**, 1031-1037 (2003).
